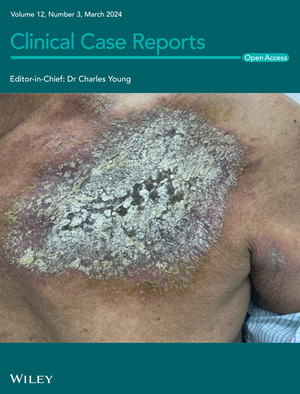# Cover Image

**DOI:** 10.1002/ccr3.8721

**Published:** 2024-03-25

**Authors:** Prajwal Pudasaini, Sadiksha Adhikari, Kinnor Das, Suraljit Gorai, Ruri D. Pamela

## Abstract

The cover image is based on the Case Report *Quack‐induced dermatitis: A case report of two dermatitis artefacta induced by quacks advise* by Prajwal Pudasaini *et al.*, https://doi.org/10.1002/ccr3.8658